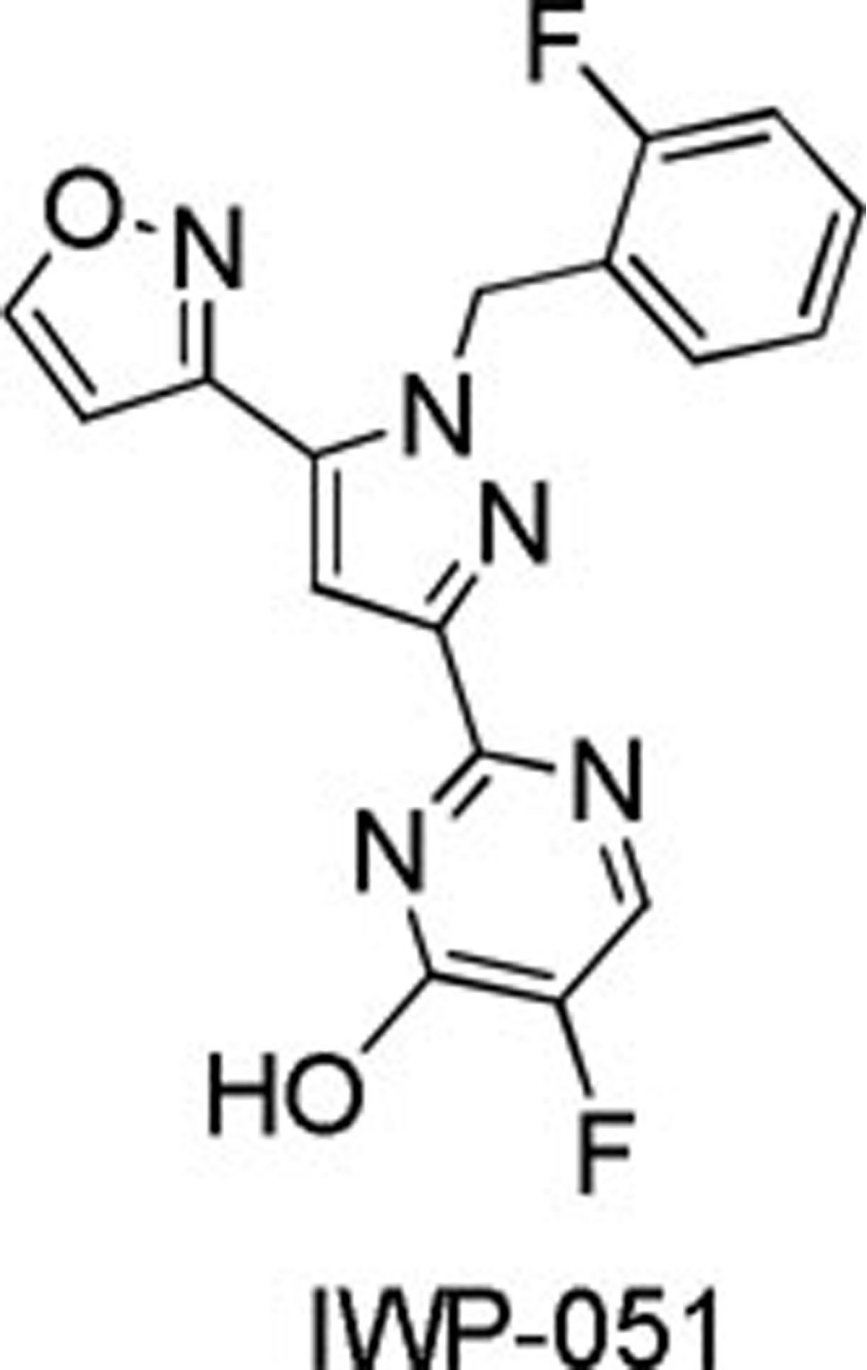# Discovery of IWP-051, a novel orally bioavailable soluble guanylate cyclase stimulator with sustained and dose-dependent hemodynamic effects

**DOI:** 10.1186/2050-6511-16-S1-A59

**Published:** 2015-09-02

**Authors:** Takashi Nakai, Nicholas R Perl, Rajesh R Iyengar, Ara Mermerian, G-Yoon J Im, Thomas W-H Lee, Glen R Rennie, James Jia, Paul A Renhowe, Timothy C Barden, James E Sheppeck, Karthik Iyer, Joon Jung, G Todd Milne, Chrissie Segal, Kimberly Long, Joy Miyashiro, Sylvie Bernier, Sarah Jacobson, Jenny Tobin, Courtney Shea, Peter Germano, Yueh-tyng Chien, Daniel Zimmer

**Affiliations:** 1 Ironwood Pharmaceuticals Inc., Cambridge, MA, USA

## Background

Soluble guanylate cyclase (sGC) stimulators are small molecule agonists of sGC that are heme-dependent, nitric oxide (NO)-independent, and act in synergy with NO. Herein, we describe a novel class of pyrazole-pyrimidine sGC stimulators and their evolution from an early lead to IWP-051 by optimizing SAR for in vitro potency, pharmacokinetic parameters, and off-target activity.

## Conclusion

IWP-051 is a potent sGC stimulator with >99% plasma protein binding, high metabolic stability, high permeability, and no efflux in a Caco-2 model of intestinal absorption. In rat PK studies, IWP-051 had low clearance and a low volume of distribution. Its elimination half-life in rats was >4 hrs. IWP-051 exhibited dose-related oral exposure, its Tmax was >3 hrs in rats, and its oral bioavailability was >40% in mice, rats, and dogs. In normotensive rats, oral doses of IWP-051 ranging from 1 to 100 mg/kg decreased mean arterial pressure in a sustained and dose-responsive manner. Distinct features of the pharmacologic profile of IWP-051, including metabolic stability, protracted gastrointestinal absorption, and sustained effect on hemodynamics upon oral dosing in rats, make IWP-051 an exciting pharmacologic advancement in the sGC stimulator class.

**Fig 1:**